# Awaiting Stress: An Interprofessional In-situ Simulation to Optimize Team Management of Cardiac Arrest in an Emergency Department Observation Unit

**DOI:** 10.5070/M5.52393

**Published:** 2026-10-31

**Authors:** Ashley Christine Rider, Michael Losak, Brooke Stimmel, Stephanie Galang, Jennifer Hunter, Kelly Roszczynialski

**Affiliations:** * Stanford University School of Medicine, Department of Emergency Medicine, Palo Alto, CA; ^ Stanford Health Care, Palo Alto, CA

## Abstract

**Audience:**

This simulation is intended for interprofessional clinical decision unit (CDU) teams.

**Introduction:**

Emergency department (ED) observation units or clinical decision units (CDU) are a common care model found in many hospitals due to their ability to provide high-quality care and reduce length of stay while also minimizing costs.^1^,^2^,^3^ Despite initial clinical stability, deterioration while in the CDU may occur. Due to the unique environment of the CDU, it is essential that the interprofessional team is prepared to manage unstable patients; yet there is minimal literature on how to best train observation unit teams.

**Educational Objectives:**

By the end of this simulation session, learners will be able to: 1) prioritize and coordinate team-based tasks within the CDU environment upon recognizing patient decompensation and cardiac arrest, 2) optimize clinical decision unit (CDU) team member roles and interprofessional communication, 3) adapt resuscitation principles in the resource-variable setting of a CDU to manage a critically ill patient.

**Educational Methods:**

This is run as an in-situ simulation with an interprofessional team as an experiential learning intervention. The in-situ format enables exploration of the team-specific and unit-specific context during the debrief.

**Research Methods:**

Participants completed a pre- and post-self-assessment via QR code along with responses to three program evaluation questions.

**Results:**

Twenty-five team members participated in six in-situ CDU simulations. On the pre-assessment, participants had a median of 4 (scale of 1- strongly disagree to 5 - strongly agree) across all categories including confidence in their role during a Code Blue, roles of team members, team priorities, trust in team members, equipment location, and comfort in providing hand-off. On the post-assessment, team priorities and trust in team members increased to a median of 5. In the program evaluation, participants found the experience valuable, particularly the debrief.

**Discussion:**

Team members started the simulation with confidence in cardiac arrest management, but participation increased their understanding of trust in team members and team priorities within the CDU context. This simulation and debrief session could help support team performance during cardiac arrest within the distinct clinical and interprofessional environment of the CDU.

**Topics:**

Simulation, Clinical Decision Unit, CDU, short stay unit, interprofessional, cardiac arrest, debriefing.

## USER GUIDE

**Table:** 

List of Resources:	
Abstract	1
User Guide	3
Instructor Materials	5
Operator Materials	12
Debriefing and Evaluation Pearls	15
Simulation Assessment	18
Appendix A: PowerPoint	23

**Learner Audience:**

Nurses, Advanced Practice Providers, techs, pharmacists, attendings, residents, any member of a team that works in the observation unit (which may involve residents or students)

**Time Required for Implementation:**

**Preparation:** 60 minutes

**Time for case:** 10 minutes

**Time for debriefing:** 15 minutes

**Recommended Number of Learners per Instructor:**

4–5 learners

**Topics:**

Simulation, Clinical Decision Unit, CDU, short stay unit, interprofessional, cardiac arrest, debriefing.

**Objectives:**

By the end of this simulation, learners will be able to:

1. Prioritize and coordinate team-based tasks within the CDU environment upon recognizing patient decompensation and cardiac arrest.
2. Optimize clinical decision unit (CDU) team member roles and interprofessional communication.
3. Adapt resuscitation principles in the resource-variable setting of a CDU to manage a critically ill patient.

### Linked objectives and methods

1. Prioritize and coordinate team-based tasks within the CDU environment upon recognizing patient decompensation and cardiac arrest: The experiential nature of in-situ simulation is the ideal setting to practice low-frequency, high-acuity events such cardiac arrest. Stakeholders from nursing, physician, and APP leadership met and developed a list of objectives, which guided the drafting of the simulation case. The case content and high-fidelity simulation allowed for the “concrete experience” phase of Kolb’s cycle, followed by reflection and abstract conceptualization offered during the debrief discussion.^4^
2. Optimize Clinical Decision Unit (CDU) team member roles and interprofessional communication: The team members performed the simulation during their shift with the same colleagues they were working with that day, representing the real interprofessional team coming together to manage a scenario and helping to enhance fidelity. During the debrief, we focused on processes and procedures specific to that team and the CDU setting.
3. Adapt resuscitation principles in the resource-variable setting of a CDU to manage a critically ill patient: The case was designed to practice Advance Cardiac Life Support (ACLS) principles within the CDU context including initial recognition of cardiac arrest by the bedside nurse and prioritization of high-quality cardiopulmonary resuscitation (CPR) and early defibrillation while balancing other roles within a small interprofessional CDU team.

### Recommended pre-reading for instructor

Familiarity with context and resources of one’s institutional CDU. For additional reading on these units, please see references 1–3 below.

### Learner responsible content (optional)

Learners should have completed ACLS.

### Results and tips for successful implementation

We held six in-situ sessions in the CDU with a total of 25 nurse, APP, technician, and pharmacist participants. Two simulation-trained physician faculty facilitated the ~15 minute debrief using the PEARLS model.^5^ The most common topics discussed in the debrief were priorities within the smaller CDU team, recruiting personnel and gathering equipment, and optimal interprofessional communication in the setting of dynamic role changes. Facilitators took notes of high-yield discussion points and summarized them in a document for email distribution to CDU staff.

Our assessment consisted of a six-question anonymous pre- and post-assessment focused on knowing roles, priorities, equipment, and trust in team members. Participants rated their agreement with each on a scale of 1 to 5, strongly disagree to strongly agree. In addition, we asked three post-participation evaluation questions.

On the pre-assessment, participants had a median of 4 (“agree”) across all statements (range 1 “strongly disagree” to 5 “strongly agree”). In the post-assessment, the median score for all participants increased to 5 (“strongly agree”) on knowing priorities during a Code Blue as well as trusting team members during a Code Blue, in line with objectives 1 and 2 for the simulation.

**Figure:**
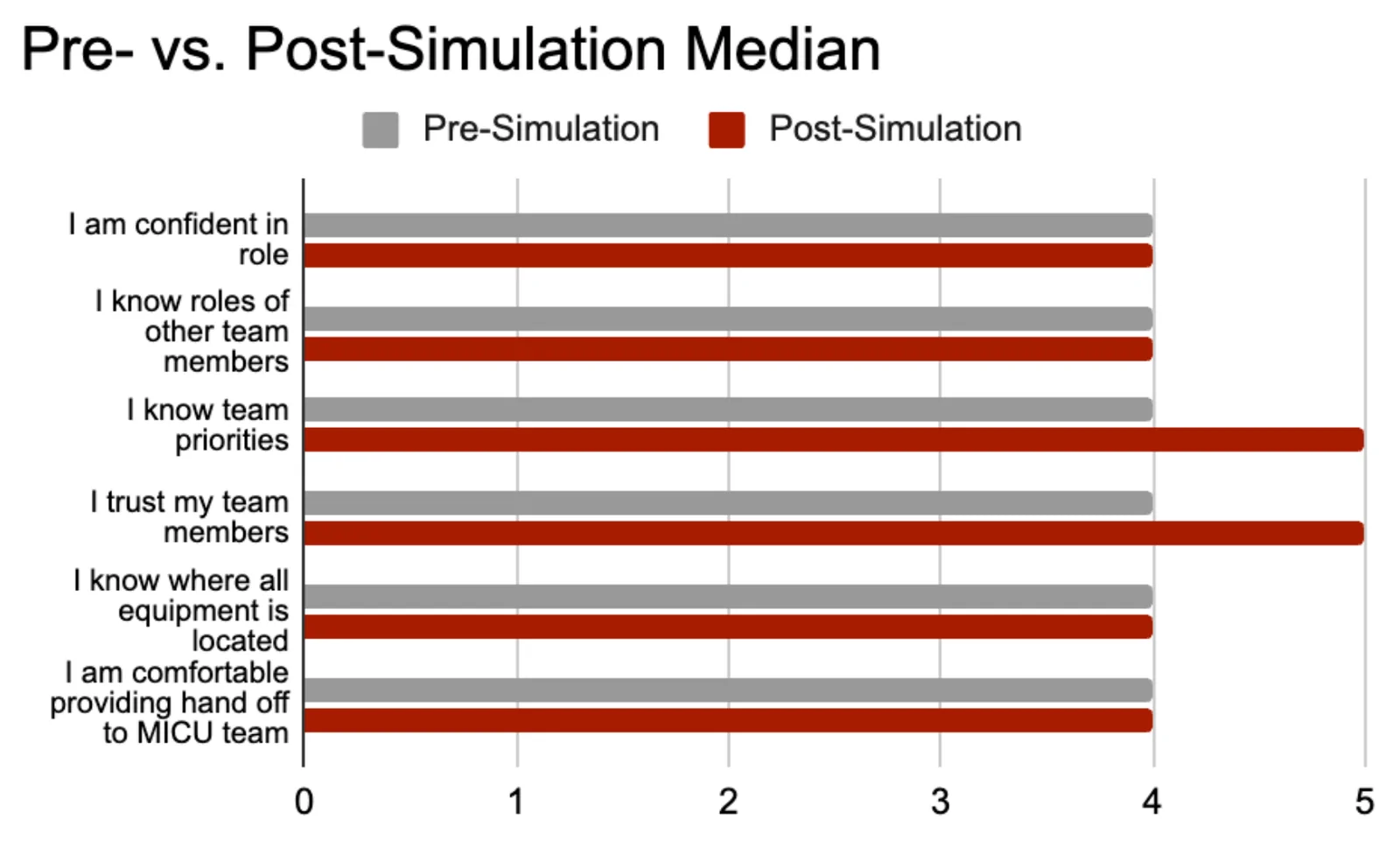


In the evaluation responses, participants reported that the most helpful aspects were the experiential nature of running the code along with the debrief discussion of challenges within the CDU and prioritization within the smaller interprofessional team of the CDU. When asked what could be improved in the future, participants reported a desire for more team-based practice opportunities.

Reflections from the facilitator team identified additional strategies for implementation. First, interprofessional partnerships were crucial in identifying optimal times for simulations and securing a room during low patient volumes. Second, facilitators needed to be flexible to accommodate both day and night shift professionals and to adjust for more pressing patient needs when they arose. Finally, there were challenges related to real-time patient care needs and availability of participants for which flexibility in timing was key.

Interprofessional teams must be prepared to manage patient deterioration in an observation unit. To optimize cardiac arrest management, health professionals must know roles, priorities, and trust their team members. Based on the pre- and post-assessment, team members had improved knowledge of team priorities as well as enhanced trust in team members after participating in the session.

### Associated content

See PowerPoint debrief

## Supplementary Information



## Figures and Tables

**Table:** 

**#1**	**History of present illness slide for participants (slide #4)**
**#2**	**Past Medical History (slide #5)**
**#3**	**Glucose (slide #7)**
**#4**	**Blood Gas (slide #8)**
**#5**	**Chemistry (slide #9)**
**#6**	**Post-Return of Spontaneous Circulation ECG (#10)**

**Table:** 

**Vitals:**	Heart Rate (HR) 58	Blood Pressure (BP) 90/50
	Respiratory Rate (RR) 18	Temperature (T) 36.8°C
	Oxygen Saturation (O_2_Sat) 94% on room air (RA)	

**Table:** 

*Glucose*	110 mg/dL

**Table:** 

*iSTAT Venous blood gas (VBG)*	
pH	7.15
pCO2	54 mmHg
pO2	40 mmHg
Lactate	5.2

**Table:** 

*iSTAT electrolytes and hemoglobin*	
Sodium	141 mEq/L
Potassium	4.7 mEq/L
Hemoglobin (Hgb)	11 g/dL

**Figure:**
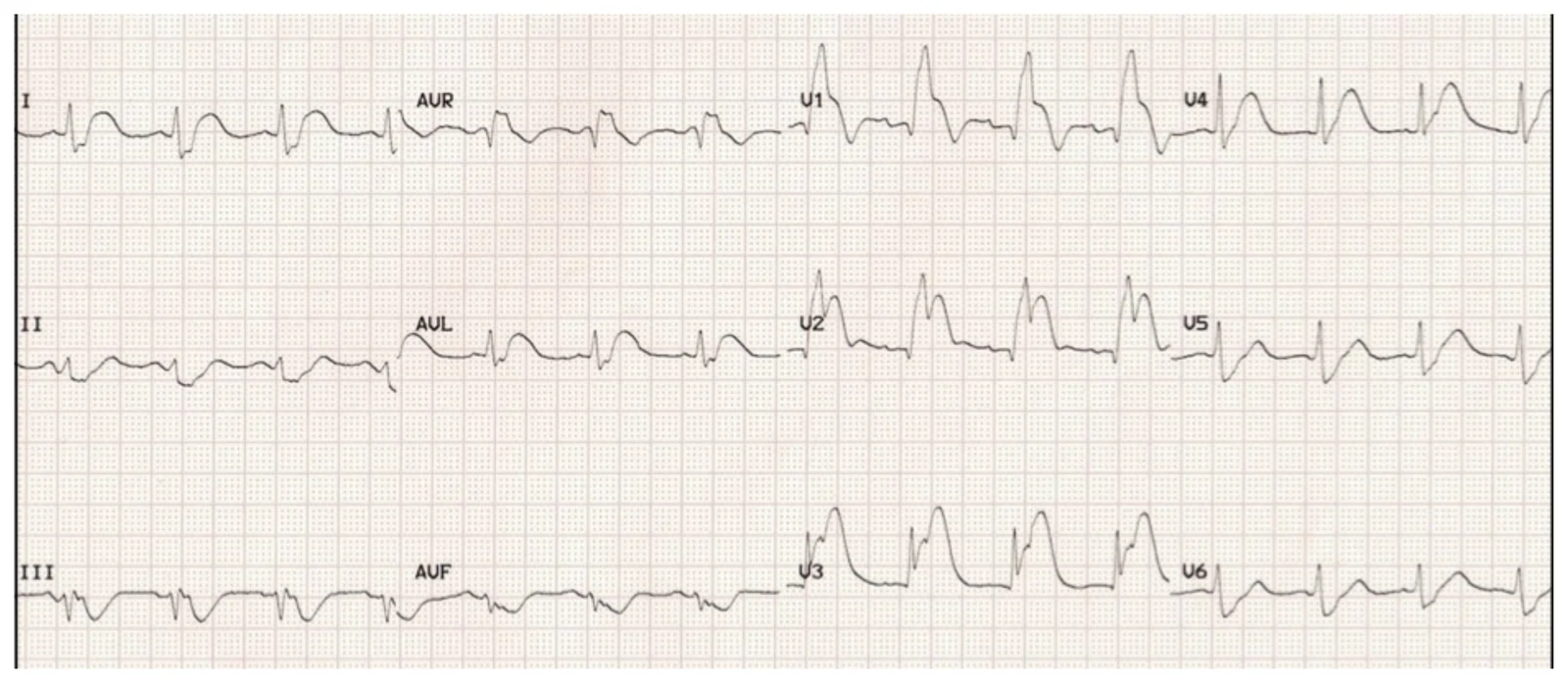


**Table:** SIMULATION EVENTS TABLE:

Minute (state)	Participant action/ trigger	Patient status (simulator response) & operator prompts	Monitor display (vital signs)
0:00 (Baseline) Stage 1 – Participants enter	RN assesses patient complaint and requests ECG	Patient is reporting severe chest pain, appears in distress. Transition to next state at 1 min mark prior to ECG.	T – 36.8° C HR - 58 BP – 90/50 RR - 18 O2 – 94%
1:00	ED tech or RN setting up for ECG	**Patient:** Loses consciousness, no longer responsive. **Learner action:** When team assesses, patient is pulseless and apneic. Team should initiate CPR, call a Code Blue, obtain code cart, assemble the team, and identify roles. **Operator prompt:** Change vital signs to be pulseless and apneic, ventricular tachycardia rhythm.	HR – 0 BP – 0 RR – 0 O2 – 70% Monitor consistent with CPR compressions
3:00 State A	First rhythm check performed.	**Patient:** continues to be pulseless and apneic. **Learner action:** Team should continue high-quality CPR while placing pads and analyzing rhythm to be ventricular tachycardia requiring defibrillation. Medications can be given and airway managed with BVM versus an advanced airway. Request glucose and iSTAT labs. **Operator prompt:** After defibrillation, rhythm changes to ventricular fibrillation-- transition to 5:00 State A.	HR – 0 BP – 0 RR – 0 O2 – 70%
3:00 State B	No pad placement or defibrillation	**Learner action:** If the patient is not defibrillated, the code continues for another round but still changes to ventricular fibrillation.	HR – 0 BP – 0 RR – 0 O2 – 70%
3:00 State C	No CPR initiated	**Learner action:** No compressions are initiated. **Operator prompt:** Trigger embedded participant to join early and give prompt to team to start compressions and transition to 5:00 State B.	HR – 0 BP – 0 RR – 0 O2 – 70%
5:00 State A	Second rhythm/pulse check performed.	**Patient:** Rhythm changes to ventricular fibrillation. **Learner action:** Team should continue high-quality CPR for 2 minutes and identify shockable rhythm and defibrillate on second pulse check/rhythm check, then resume CPR. **Operator prompt:** After second defibrillation ROSC is obtained.	HR – 0 BP – 0 RR – 0 O2 – 70%
5:00 State B	ICU embedded participant guides team in resuscitation	**Patient:** Rhythm changes to ventricular fibrillation. Embedded participant will enter and alert the team to the shockable rhythm. **Learner action:** Defibrillates patient after guidance from embedded participant. Operator Prompt: After this single defibrillation patient will achieve ROSC and transition to state 7:00.	HR – 0 BP – 0 RR – 0 O2 – 70%
7:00	Participants recognize ROSC.	**Patient:** ROSC is obtained but patient remains minimally responsive. ECG shows STEMI. **Learner action:** Initiate post-ROSC care. Fluids/pressors initiated for hypotension. Consideration of intubation if advanced airway not in place. ECG obtained, shows STEMI. Heart alert activated. Handoff to ICU team and cardiologist. **Operator prompt:** Display ECG when indicated. If ROSC is not recognized, the patient begins to moan and move.	T - 36.8° C HR - 62 BP – 80/40 RR - 4 O2- 90%
9:00–10:00	Case concludes	Embedded participants arrive and receive handoff from CDU care team and take over management.	T – 36.8° C HR – 62 BP – 80/40 RR - 4 O2 – 90%

**Table:** 

**Critical Actions:** Prioritize tasks: Activate a Code Blue Prioritize tasks: Initiate CPR Team Communication: Assemble the team Team Communication: Appropriately assume roles and tasks Prioritize tasks: Bring code cart to bedside Prioritize tasks: Place pads Principles of ACLS: Deliver high-quality CPR Principles of ACLS: Identify and defibrillate a shockable rhythm Principles of ACLS: Locate items within code cart Principles of ACLS: Deliver breaths by BVM Principles of ACLS: Administer medications Team Communication: Identify underlying etiology of the arrest and provide handoff	0:00

**Table:** 

	Milestone	Did not achieve level 1	Level 1	Level 2	Level 3
1	**Emergency Stabilization (PC1)**	□ Did not achieve Level 1	□ Recognizes abnormal vital signs	□ Recognizes an unstable patient, requiring intervention Performs primary assessment Discerns data to formulate a diagnostic impression/plan	□ Manages and prioritizes critical actions in a critically ill patient Reassesses after implementing a stabilizing intervention
2	**Performance of focused history and physical (PC2)**	□ Did not achieve Level 1	□ Performs a reliable, comprehensive history and physical exam	□ Performs and communicates a focused history and physical exam based on chief complaint and urgent issues	□ Prioritizes essential components of history and physical exam given dynamic circumstances
3	**Diagnostic studies (PC3)**	□ Did not achieve Level 1	□ Determines the necessity of diagnostic studies	□ Orders appropriate diagnostic studies. Performs appropriate bedside diagnostic studies/procedures	□ Prioritizes essential testing Interprets results of diagnostic studies Reviews risks, benefits, contraindications, and alternatives to a diagnostic study or procedure
4	**Diagnosis (PC4)**	□ Did not achieve Level 1	□ Considers a list of potential diagnoses	□ Considers an appropriate list of potential diagnosis May or may not make correct diagnosis	□ Makes the appropriate diagnosis Considers other potential diagnoses, avoiding premature closure
5	**Pharmacotherapy (PC5)**	□ Did not achieve Level 1	□ Asks patient for drug allergies	□ Selects an medication for therapeutic intervention, consider potential adverse effects	□ Selects the most appropriate medication and understands mechanism of action, effect, and potential side effects Considers and recognizes drug-drug interactions
6	**Observation and reassessment (PC6)**	□ Did not achieve Level 1	□ Reevaluates patient at least one time during case	□ Reevaluates patient after most therapeutic interventions	□ Consistently evaluates the effectiveness of therapies at appropriate intervals
7	**Disposition (PC7)**	□ Did not achieve Level 1	□ Appropriately selects whether to admit or discharge the patient	□ Appropriately selects whether to admit or discharge Involves the expertise of some of the appropriate specialists	□ Educates the patient appropriately about their disposition Assigns patient to an appropriate level of care (ICU/Tele/Floor) Involves expertise of all appropriate specialists
9	**General Approach to Procedures (PC9)**	□ Did not achieve Level 1	□ Identifies pertinent anatomy and physiology for a procedure Uses appropriate Universal Precautions	□ Obtains informed consent Knows indications, contraindications, anatomic landmarks, equipment, anesthetic and procedural technique, and potential complications for common ED procedures	□ Determines a back-up strategy if initial attempts are unsuccessful Correctly interprets results of diagnostic procedure
20	**Professional Values (PROF1)**	□ Did not achieve Level 1	□ Demonstrates caring, honest behavior	□ Exhibits compassion, respect, sensitivity and responsiveness	□ Develops alternative care plans when patients’ personal beliefs and decisions preclude standard care
22	**Patient centered communication (ICS1)**	□ Did not achieve level 1	□ Establishes rapport and demonstrates empathy to patient (and family) Listens effectively	□ Elicits patient’s reason for seeking health care	□ Manages patient expectations in a manner that minimizes potential for stress, conflict, and misunderstanding. Effectively communicates with vulnerable populations, (at risk patients and families)
23	**Team management (ICS2)**	□ Did not achieve level 1	□ Recognizes other members of the patient care team during case (nurse, techs)	□ Communicates pertinent information to other healthcare colleagues	□ Communicates a clear, succinct, and appropriate handoff with specialists and other colleagues Communicates effectively with ancillary staff

**Figure:**
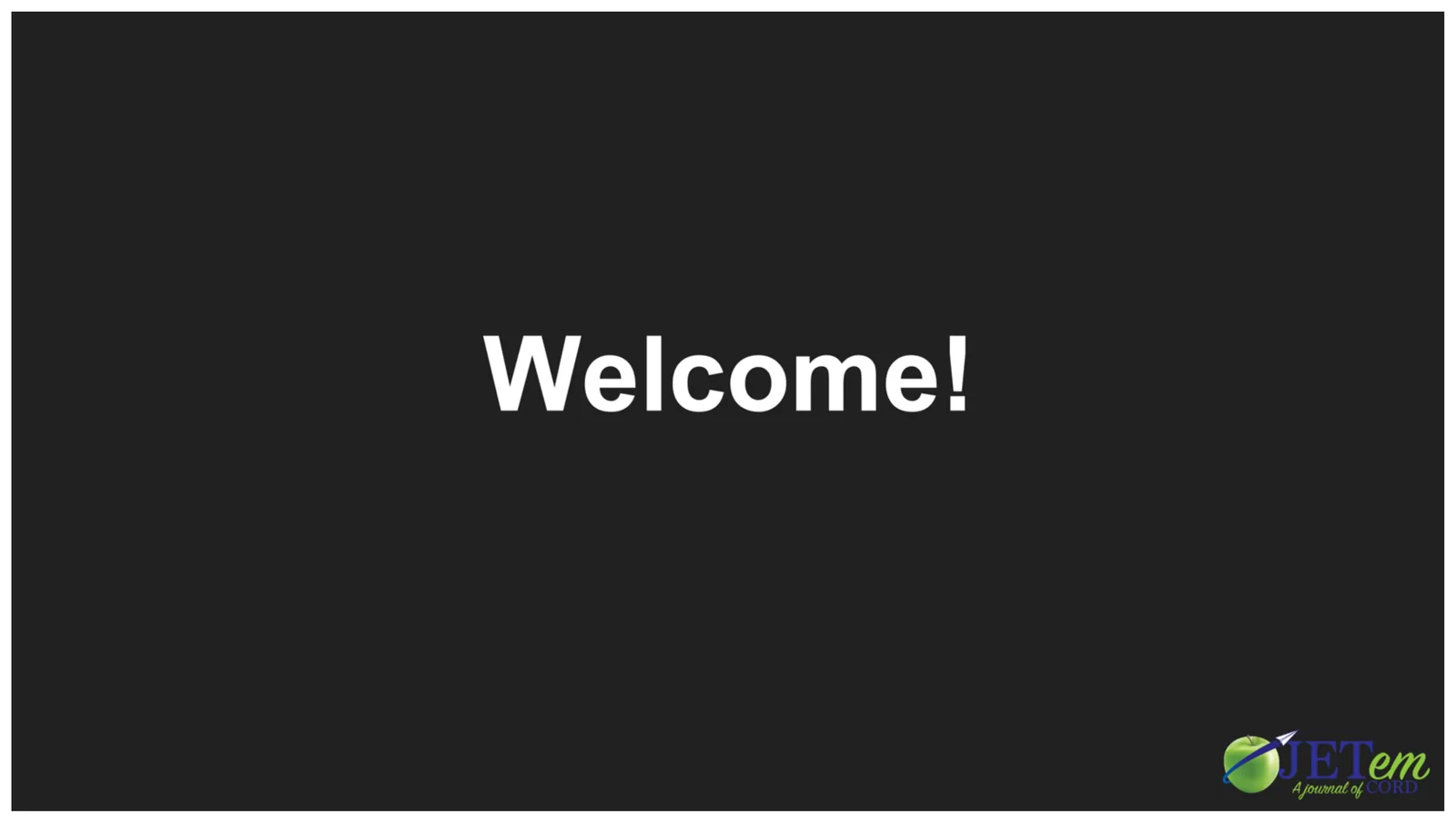
Please see associated PowerPoint file

## INSTRUCTOR MATERIALS

**Case Title:** Awaiting Stress

**Case Description & Diagnosis (short synopsis):** Patient is a 62-year-old female with history of hypertension (HTN), hyperlipidemia (HLD), diabetes (DM), and rheumatoid arthritis (RA) who presented to the emergency department (ED) earlier today with two episodes of chest pain this week. Her electrocardiogram (ECG) did not show any new ischemic changes. Troponins were in the indeterminate range and stable. She was placed in the CDU for a stress test in the morning. At 6:45 AM (day shift) or 10:00 PM (night shift), she began complaining of severe chest pain. She develops bradycardia and then loses pulses. She is in ventricular tachycardia followed by ventricular fibrillation. Return of spontaneous circulation is obtained after six minutes of Advanced Cardiac Life Support (ACLS).

**Equipment or Props Needed:**

In-situ – the case must occur in the CDU or equivalent observation unit

Other equipment

- A high-fidelity mannequin and a monitor with real time vitals; note that a low-fidelity mannequin may also be used
- Zoll monitor/defibrillator
- Defibrillator pads
- Intravenous (IV) start kit
- Glucometer
- Mock medication vials including epinephrine, lidocaine, bicarbonate, calcium, normal saline or Lactated Ringer’s
- Laryngoscope
- Bag Valve Mask (BVM)
- Laryngeal Mask Airway (LMA)
- Endotracheal tube and stylet
- 10 mL syringe
- The mannequin was sitting up on a hospital gurney in a hospital gown with an IV in place at the start of the scenario. Telemetry monitoring was ongoing. No moulage needed.

**Embedded participants needed:**

The facilitator provides the voice of the mannequin. There is only one embedded participant who enters at the end of the scenario and plays the role of the intensive care unit (ICU) team member and/or the interventional cardiologist.

**Stimulus Inventory:**

**Background and brief information:** Patient is a 62-year-old F with history of HTN, HLD, DM, rheumatoid arthritis (RA) who presented to the ED earlier today with two episodes of chest pain this week. Her ECG did not show any new ischemic changes. Troponins were in the indeterminate range and stable. She was placed in the CDU for a stress test in the morning.

**Initial presentation:** The patient is lying in a CDU bed, complaining of chest pain. She is being assessed by a single bedside nurse.

**How the scenario unfolds:** At 6:45 AM (day shift) or 10:00 PM (night shift), she began complaining of severe chest pain. The bedside nurse approaches her at bedside and performs an initial assessment. Patient will be in distress secondary to pain but talking. Vital signs will show borderline hypotension. An ECG is requested, but patient will become bradycardic, unresponsive and arrest before an ECG can be obtained. The bedside RN will assess for pulses and initiate high-quality CPR while calling for a Code Blue. Other team members will come to bedside, and one will bring the code cart. Priority roles will be determined among the team members, including CPR, airway management, medication administration, Zoll operator, timekeeper, and team lead. CPR will be continued, and pads will be applied for rhythm analysis. The first rhythm will be identified as ventricular tachycardia which will be defibrillated by the team. The patient will remain pulseless and apneic. The subsequent rhythm check will reveal ventricular fibrillation, which will also be defibrillated. At minute 6 after arrest, patient will have Return Of Spontaneous Circulation (ROSC). Initial vital signs will reveal hypotension, which will be treated with fluids and consideration of pressors. The patient will remain unresponsive with shallow breaths, requiring assisted breaths with BVM if no advanced airway in place. Team will obtain post-ROSC ECG which will show a ST-elevation myocardial infarction (STEMI) and a Heart Alert is Activated. Hand-off will be provided to a responding interventional cardiologist and/or the ICU team member.

**Critical actions:**

1. Prioritize tasks: Activate a Code Blue
2. Prioritize tasks: Initiate CPR
3. Team Communication: Assemble the team
4. Team Communication: Appropriately assume roles and tasks
5. Prioritize tasks: Bring code cart to bedside
6. Prioritize tasks: Place pads
7. Principles of ACLS: Deliver high-quality CPR
8. Principles of ACLS: Identify and defibrillate a shockable rhythm
9. Principles of ACLS: Locate items within code cart
10. Principles of ACLS: Deliver breaths by BVM
11. Principles of ACLS: Administer medications
12. Team Communication: Identify underlying etiology of the arrest and provide handoff

**Case Title:** Awaiting Stress

**Chief Complaint:** chest pain

**General Appearance:** uncomfortable

**Primary Survey:**

- **Airway:** speaking
- **Breathing:** bilateral clear breath sounds
- **Circulation:** thready pulses, cool and clammy

**History:**

- **History of present illness:** Patient is a 62-year-old female with history of HTN, HLD, T2DM, rheumatoid arthritis who presented to the ED earlier today with two episodes of chest pain (CP) this week, one 3 days ago that lasted 20 minutes and resolved, another one this morning that lasted 15 minutes and resolved. Her ECG in the ED did not show any new ischemic changes. Troponins were in the indeterminate range and stable. No recurrence of CP while in the ED. Other labs within normal limits. She was placed in the CDU for a stress test in the morning. At 6:45 AM (day shift) or 10:00 PM (night shift) she began complaining of severe chest pain.
- **Past medical history:** hypertension, hyperlipidemia, type II diabetes mellitus, rheumatoid arthritis
- **Past surgical history: N/A**
- **Patient’s medications:** Metformin, atorvastatin, lisinopril, methotrexate
- **Allergies:** Penicillin
- **Social history:** Lives with family, no tobacco, no alcohol, no other substances
- **Family history:** Mother with CAD in 60s

**Secondary Survey/Physical Examination:**

- **General appearance:** In acute distress, uncomfortable appearing
- **HEENT:** Normal
- **Neck:** Normal
- **Heart:** regular rate and rhythm
- **Lungs:** Crackles at the bases
- **Abdominal/GI:** Non-distended, non-tender
- **Neuro:** A&O x3, moving all extremities
- **Skin:** Cool, clammy, no color changes, 20-gauge needle in the left antecubial fold
- **Psych:** Anxious

*Post-ROSC ECG*

Anterior Myocardial Infarction. Example 8. Burns E, Buttner R. In: Life In the Fast Lane. https://litfl.com/anterior-myocardial-infarction-ecg-library/ October 8, 2024. CC BY-NC-SA 4.0.

**Diagnosis:** cardiac arrest secondary to STEMI

**Disposition:** cath lab followed by ICU-Level care

## DEBRIEFING AND EVALUATION PEARLS

*Awaiting Stress*

**Facilitator Guide to Debriefing:**

The debrief should open with a reactions phase to understand the learner’s experience. After summarizing the case, the debrief should focus on three main topics, aligned with the learning objectives: (1) coordinating the team response, (2) optimizing roles and communication, and (3) adapting the resuscitation to the CDU. The discussion should emphasize the unique approach to each of these topics in the CDU, where resources, staffing, and familiarity with resuscitation may vary.

First, the learners should develop an approach to coordinate a team response to a cardiac arrest in the unique setting of the CDU. Unlike traditional ED resuscitation bays, the initial responder may be a single bedside nurse in the room with the patient, making early recruitment of additional personnel critical. Facilitators should review local escalation workflows (eg, activating a code blue button, notifying the charge nurse), and outline warning signs to recognize impending decompensation, such as chest pain, tachypnea, diaphoresis, and unstable vitals that warrant escalation. The facilitator should also discuss the location of specific items in the CDU to facilitate cardiac arrest care including a backboard, stool, and a crash cart. Immediate initiation of CPR is of the highest priority and should be started by the bedside team member while activating this chain. CPR within two minutes is associated with lower mortality for in-hospital arrest.^7^

Second, this case emphasizes explicit role assignment and structured communication as core components of effective interprofessional teamwork in the CDU. Clear role designation improves team efficiency and reduces delays in critical interventions.^8^,^9^ In smaller CDU teams, these roles may need to be distinctly prioritized, combined, or reassigned dynamically. In small teams of two individuals, we discussed prioritization of CPR and preparation for early defibrillation, while simultaneously running the code, followed by airway management and medication administration as more team members become available.^10^

Closed-loop communication should be emphasized as a foundational teamwork skill. This involves a directed request to a specific team member, acknowledgment of the task, and confirmation upon completion. This strategy, central to TeamSTEPPS, reduces errors and improves task completion in high-acuity settings.^11^ This is especially important during dynamic role changes of a small team, and therefore this skill should be explicitly practiced and discussed in the debrief. Facilitators should also introduce the TeamSTEPPS concepts of situation monitoring, cross-monitoring, and shared mental models to emphasize the importance of situational awareness and alignment among teams with varying Levels of resuscitation experience.

**Other implementation and debriefing tips:**

Advanced questions may arise regarding the management of a critically ill patient. Given the limited debrief time frame due to the in-situ and on-shift nature of this case, we recommend focusing on the interprofessional team management objectives for this case and encouraging individualized follow-up for more specific questions.

**Pearls:**

**Objective 1: Prioritize and coordinate team-based tasks**

- **Recognition of decompensation and cardiac arrest:** Noting cardiopulmonary and neurologic changes (decreased responsiveness, diaphoresis, pallor, change in rhythm on telemetry, hypotension, change or loss of respiratory rate). Assessing the patient and checking central pulses.^12^
- **Mobilizing team:** Review the unit-specific processes to call for additional resources.
- **Task prioritization:** For a smaller team, priorities include starting high-quality CPR, recruiting help, obtaining additional equipment, and defibrillating a shockable rhythm.

**Objective 2: Optimizing team member roles and communication**

- **Roles:** Clearly establish roles once more help arrives: code leader, CPR rotation, airway (BVM), medication administration, timer, and recorder.
- **Communication:** Provide a shared mental model and verbalize priorities. Ensure closed-loop communication.

**Objective 3. Adapt resuscitation principles in a resource-variable setting**

- **Manage a critically ill patient using ACLS principles:** Review steps to ensure high quality of compressions (putting the head of bed down, putting down the side rail, placing patient on the back board, using a stool, rotating compressors, minimizing interruptions, ensuring adequate rate and depth, and monitoring CPR quality on Zoll). Perform a rhythm/pulse check every 2 minutes. Identify shockable rhythm on rhythm/pulse check and promptly defibrillate. Ensure team clears from the bed prior to defibrillation for safety and immediately resume compressions afterwards. Ventilate the patient using a BVM or advanced airway. Give epinephrine in all adult cardiac arrest cases every 3–5min. Give amiodarone or lidocaine for shockable rhythms. Identify and treat reversible causes. Review components of post-ROSC care including stabilization measures for both the airway and hemodynamics and a post-ROSC ECG to evaluate need for cardiac catheterization.^12^
- **Adapting resuscitation to CDU:** Discuss specific priorities within a smaller team, activation protocols, and the location of equipment within the CDU.

**Wrap Up:**

There is no formal lecture following the simulation case; however, we recommend that facilitators thank participants for joining, invite any additional questions about patient management, and subsequently distribute learning points discussed during the debriefs to CDU team members.

## SIMULATION ASSESSMENT

*Awaiting Stress*

Learner: _________________________________________

***Assessment Timeline***

This timeline is to help observers assess their learners. It allows observer to make notes on when learners performed various tasks, which can help guide debriefing discussion.

**Critical Actions:**

□ Prioritize tasks: Activate a Code Blue
□ Prioritize tasks: Initiate CPR
□ Team Communication: Assemble the team
□ Team Communication: Appropriately assume roles and tasks
□ Prioritize tasks: Bring code cart to bedside
□ Prioritize tasks: Place pads
□ Principles of ACLS: Deliver high-quality CPR
□ Principles of ACLS: Identify and defibrillate a shockable rhythm
□ Principles of ACLS: Locate items within code cart
□ Principles of ACLS: Deliver breaths by BVM
□ Principles of ACLS: Administer medications
□ Team Communication: Identify underlying etiology of the arrest and provide handoff

**Summative and formative comments:**

**Milestones assessment:**
